# Supplementary figures and images for: A model explaining mRNA level fluctuations based on activity demands and RNA age
Source: PLoS Comput Biol. 2021 Jul 23;17(7):e1009188. doi: 10.1371/journal.pcbi.1009188 (PMC8336849; doi:10.1371/journal.pcbi.1009188)

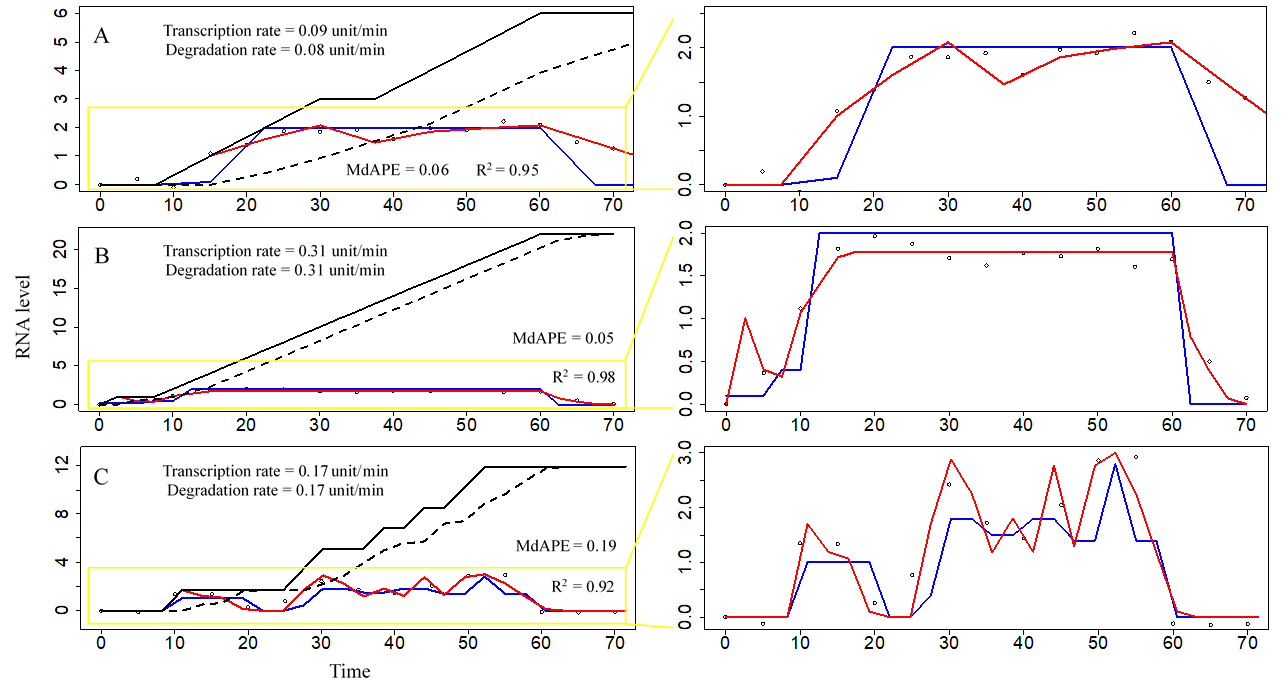

Supplement: S1 Fig — Hollow circles represent RNA levels of experimental data obtained from the literature [21]. Blue line represents the demand for RNA activity (DRA). Red curve represents the RNA level based on the results of the model simulation. Black solid line represents the cumulative transcription RNA level calculated by the model. Black dashed line represents the accumulated degradation RNA level calculated by the model. The x-axis is time, and the unit of time is minute. The y-axis is the RNA level, and the unit is normalized fold change of mRNA level with the baseline subtracted. (A) Partially regular fluctuations: analysis of mRNA level fluctuations of the Saccharomyces cerevisiae GRX1 gene by the model. The estimated results of the parameters: accumulated transcription level = 6 units, accumulated RNA degradation level = 4.7 units, life span = 30 minutes, pulse transcription level at each time = 1 unit, the rate of survival at RNA age 1 = 0.6, the survival rate at RNA age 2 = 0.8, and the survival rate at RNA age 3 = 0.8. (B) Regular fluctuations: analysis of mRNA level fluctuations of the S. cerevisiae NTH1 gene by the model. The estimated results of the parameters: accumulated transcription level = 22 units, accumulated RNA degradation level = 22 units, life span = 10 minutes, pulse transcription level at each time = 1 unit, the rate of survival at RNA age 1 = 0.4, the survival rate of RNA age 2 = 0.8, and the survival rate of RNA age 3 = 0.2. (C) Irregular fluctuations: analysis of mRNA level fluctuations of the S. cerevisiae CYB2 gene by the model. The estimated results of the parameters: accumulated transcription level = 11.9 units, accumulated RNA degradation level = 11.9 units, life span = 11 minutes, pulse transcription level at each time = 1.7 units, the rate of survival at RNA age 1 = 0.7, the survival rate of RNA age 2 = 0.9, and the survival rate of RNA age 3 = 0.1. (TIF) [file pcbi.1009188.s001.tif]
