# Supplementary material for: A model explaining mRNA level fluctuations based on activity demands and RNA age
Source: PLoS Comput Biol. 2021 Jul 23;17(7):e1009188. doi: 10.1371/journal.pcbi.1009188 (PMC8336849; doi:10.1371/journal.pcbi.1009188)
Supplement: S1 Table — (PDF) [file pcbi.1009188.s002.pdf]

S1 Table. Experiment data of single cell RNA abundance

Gene name	Experiment time	RNA abundance*	References
CYB2 (*Saccharomyces cerevisiae*)	(Unit: min) 0, 5, 10, 15, 20, 25, 30, 35, 40, 45, 50, 55, 60, 65, 70	(Standardized unit) 0, -0.11, 1.35, 1.34, 0.26, 0.77, 2.43, 1.73, 1.44, 2.05, 2.87, 2.93, -0.11, -0.15, -0.12	Hao and O'Shea 2011 [1]
GRX1 (*S. cerevisiae*)	(Unit: min) 0, 5, 10, 15, 20, 25, 30, 35, 40, 45, 50, 55, 60, 65, 70	(Standardized unit) 0, 0.19, -0.11, 1.08, 1.38, 1.87, 1.86, 1.92, 1.60, 1.97, 1.92, 2.21, 2.09, 1.50, 1.26	Hao and O'Shea 2011 [1]
HSP26 (*S. cerevisiae*)	(Unit: min) 0, 5, 10, 15, 20, 25, 30, 35, 40, 45, 50, 55, 60, 65, 70	(Standardized unit) 0, -0.1, 0, 1.12, 3.02, 3.47, 3.14, 2.91, 3.29, 3.16, 3.26, 2.89, 3.16, 1.57, 1.01	Hao and O'Shea 2011 [1]
NTH1 (*S. cerevisiae*)	(Unit: min) 0, 5, 10, 15, 20, 25, 30, 35, 40, 45, 50, 55, 60, 65, 70	(Standardized unit) 0, 0.36, 1.12, 1.82, 1.97, 1.88, 1.71, 1.62, 1.77, 1.73, 1.82, 1.61, 1.70, 0.50, 0.07	Hao and O'Shea 2011 [1]
YNR014W (*S. cerevisiae*)	(Unit: min) 0, 5, 10, 15, 20, 25, 30, 35, 40, 45, 50, 55, 60, 65, 70	(Standardized unit) 0, 2.78, 1.19, 5.78, 1.07, 2.81, 0.77, 2.32, 0.51, 2.48, 0.30, 2.42, 0.30, -0.6, -0.54	Hao and O'Shea 2011 [1]
GnRH (Mouse GT1-1 cell)	(Unit: h) 0.5, 1, 1.5, 2, 2.5, 3, 3.5, 4, 4.5, 5, 5.5, 6, 6.5, 7, 7.5, 8, 8.5, 9, 9.5, 10, 10.5, 11, 11.5, 12, 12.5, 13, 13.5, 14, 14.5	(Unit: normalized photonic emissions)) 100, 89, 88.5, 92.5, 96.5, 117.5, 103, 120.5, 140, 132.5, 138, 122, 138.5, 129, 128.5, 134.5, 141.5, 154, 171, 154, 138, 138.5, 124.5, 147.5, 138.5, 157, 170, 167.5, 173	Nuñez et al. 1998 [2]

*RNA abundance values were calculated by drawing grids to digitize the original Figs. The data of CRX1 and HSP26 represent partially regular fluctuations in gene expression, the data of NTH1 and YNR014W represent regular fluctuations in gene expression, and the data of CYB2 and GnRH represent irregular fluctuations in gene expression.

Reference

1. Hao N, O'Shea EK. Signal-dependent dynamics of transcription factor translocation controls gene expression. Nat Struct Mol Biol. 2011; 19: 31-39.

2. Nuñez L, Faught WJ, Frawley LS. Episodic gonadotropin-releasing hormone gene expression revealed by dynamic monitoring of luciferase reporter activity in single, living neurons. Proc Natl Acad Sci USA. 1998; 95: 9648-9653.
