# Supplementary material for: A model explaining mRNA level fluctuations based on activity demands and RNA age
Source: PLoS Comput Biol. 2021 Jul 23;17(7):e1009188. doi: 10.1371/journal.pcbi.1009188 (PMC8336849; doi:10.1371/journal.pcbi.1009188)
Supplement: S2 Table — (PDF) [file pcbi.1009188.s003.pdf]

S2 Table. A set of parameter values used in the model to simulate RNA level fluctuations

	Type	RNA age										
		0	1	2	3	4	5	6	7	8	9	10
RNA activity coefficient	A	1	1	1	1	1	1	1	1	1	1	1
	B	1	0.9	0.8	0.7	0.6	0.5	0.4	0.3	0.2	0.1	0
	C	0	0.2	0.4	0.6	0.8	1	0.8	0.6	0.4	0.2	0
	D	0	0.1	0.2	0.3	0.4	0.5	0.6	0.7	0.8	0.9	1
RNA survival rate	A	0.5	0.5	0.5	0.5	0.5	0.5	0.5	0.5	0.5	0.5	0
	B	1	0.9	0.8	0.7	0.6	0.5	0.4	0.3	0.2	0.1	0
	C	0.1	0.2	0.4	0.6	0.8	1	0.8	0.6	0.4	0.2	0
RNA level at age 0[a] (Pulsing transcription)	Unregulated transcription: 100 per time with no limit imposed by the DRA.											
	If TRA<DRA, then the RNA level at age 0=100; else RNA level at age 0=0.											
DRA	Stable values: 50, 80, 150, 400											
	Cycling change: alternating between 50 and 150											
	Aperiodic change: $DRA=400/(1+e^{4-0.2*time})$											

[a]TRA is total RNA activity at all RNA ages in the cell. DRA is the demand for RNA activity.
