# Supplementary material for: A model explaining mRNA level fluctuations based on activity demands and RNA age
Source: PLoS Comput Biol. 2021 Jul 23;17(7):e1009188. doi: 10.1371/journal.pcbi.1009188 (PMC8336849; doi:10.1371/journal.pcbi.1009188)
Supplement: S3 Table — (PDF) [file pcbi.1009188.s004.pdf]

S3 Table. Parameter values in the model used to simulate the relationship between RNA and protein level fluctuations

	Age of protein or RNA[a]										
	0	1	2	3	4	5	6	7	8	9	10
RNA activity coefficient	0	0.6	0.7	0.8	0.9	1	0.9	0.8	0.7	0.6	0
RNA survival rate	1	0.9	0.8	0.7	0.6	0.5	0.4	0.3	0.2	0.1	0
RNA level at age 0[b]	If TRA < DRA, then RNA level at age 0=100; else RNA level at age 0=0.										
DRA[c]	If TPA < DPA, DRA= (DPA-TPA) × 2; else DRA=0.										
Protein level/RNA activity[d]	1										
Protein activity coefficient	0	0.6	0.7	0.8	0.9	1	0.9	0.8	0.7	0.6	0
Protein survival rate	1	0.9	0.8	0.7	0.6	0.5	0.4	0.3	0.2	0.1	0
Protein level at age 0	If TPA < DPA and TRA ≥ 50, protein level at age 0=50; if TPA < DPA and TRA< 50, protein level at age 0=TRA; if TPA ≥ DPA, protein level at age 0=0.										
DPA	To show fluctuations in both RNA and protein levels: DPA=25										
	To show fluctuations in RNA levels and stable trends in protein levels: DPA=175										
	To show the stable trends of both RNA and protein levels: DPA=200										
	Cycling change: DPA alternating between 50 and 150										

[a] Protein lifespan and age classification are set to be equal to the corresponding values for RNA.

[b]TRA is total RNA activity at all RNA ages. DRA is the demand for RNA activity.

[c]TPA is total protein activity at all protein ages. DPA is the demand for protein activity. The settings for DRA depend on the values of protein level/RNA activity ratios, protein survival rates, and protein activity coefficients. If TPA < DPA, protein activity at protein age 1 is the first to respond to DPA shortage because only protein at age 0 can be produced by translation, and the protein activity coefficient at protein age 0 is 0. The relationships between DRA, DPA, and TPA are derived as follows:

$$\mathrm{DPA} - \mathrm{TPA} = \text{level of protein at age 0 required} \times \text{protein survival rate at age 0} \times \text{protein survival rate at age 1} \times \text{protein activity coefficient at age 1}$$

$$\mathrm{DPA} - \mathrm{TPA} = \mathrm{DRA} \times (\text{protein level/RNA activity constant}) \times \text{protein survival rate at age 0} \times \text{protein survival rate at age 1} \times \text{protein activity coefficient at age 1}$$

$$\mathrm{DPA} - \mathrm{TPA} = \mathrm{DRA} \times 1 \times 1 \times 0.9 \times 0.6$$

$$\mathrm{DRA} \approx (\mathrm{DPA} - \mathrm{TPA}) \times 2$$

[d]This means that 1 unit of RNA activity can be transformed into 1 unit of protein level.
