# Supplementary material for: A model explaining mRNA level fluctuations based on activity demands and RNA age
Source: PLoS Comput Biol. 2021 Jul 23;17(7):e1009188. doi: 10.1371/journal.pcbi.1009188 (PMC8336849; doi:10.1371/journal.pcbi.1009188)
Supplement: Attachment — Submitted filename: 2021-6-8-responses to the reviewers.docx [file pcbi.1009188.s007.docx]

Dear Dr. Zou and reviewers,

We appreciate the comments from the reviewers of our manuscript. The suggestions were quite helpful. We want to extend our appreciation to you for taking the time and effort necessary to provide such insightful guidance.

We have revised the manuscript addressing the reviewers' comments. The changes to the original manuscript are marked in red in the text. The axes of the figures have been labeled. Figure 7a has been revised to include larger fonts. Figure S1 and R scripts for plotting have been added as supplementary materials. Itemized responses to your comments and suggestions are as follows:

**Reviewer #1**

**1) The authors developed a simulation model based on activity demands and RNA age to explore the mechanisms of RNA level fluctuations. The model used single-cell time series gene expression experimental data to determine the model parameters. The model was also able to predict RNA levels under simulation backgrounds, such as oscillation in RNA abundance, stable RNA levels and the relationship between RNA and protein levels and metabolic rates. The manuscript may benefit from the following revisions.**

Our response: Thank you for the comments.

**2)** **1. The axes of the figures need to be labeled** 

Our response: Thank you for the comment. We have labeled the axes of the figures in the revised version.

**3) 2. How are the parameters for the two-unit model that’s used to explain the relationship between RNA and protein dynamics determined?**

Our response: Thank you for the comment. A two-unit model was used to explain the relationships between RNA and protein dynamics via the simulation background method. Cycling and stable levels are regular changes that are easy to explain; hence, we simulated the cycling and stable levels of RNA and protein. According to the parameter sets for cycling RNA levels (Figure 2) and stable RNA levels (Figure 4), we designed the parameters to simulate three categories of the relationships between RNA and protein levels: cycling RNA levels and cycling protein levels, cycling RNA levels and stable protein levels, and stable RNA levels and stable protein levels. These three categories of the relationships between RNA and protein levels were reported in a review (Liu et al., 2016).

[1] Liu Y, Beyer A, Aebersold R. 2016. On the dependency of cellular protein levels on mRNA abundance. *Cell* 165: 535-550.

**4) Is there any difference with the exhaustive search method used for the RNA level model?**

Our response: Thank you for the comment. An exhaustive search method can be used in an RNA level model. The exhaustive search method used for a one-unit RNA level model in Figure 1 simulated real experimental data. We set the value range of specific parameters according to detailed experimental values and then used the exhaustive search method to select a set of parameters that provided a good fit for experimental values. A two-unit model simulating the relationships between RNA and protein levels in Figure 5 shows a study of simulation background, in which parameters were designed before the simulation.

**5) 3. Figure 7 needs more elaboration in the main text.**

Our response: Thank you for the comment. We have added additional explanations of Figure 7 in the revised manuscript. The levels and total activities of a specific biomolecule are influenced by other biomolecules and in turn regulate other biomolecules, forming a biomolecular relationship chain or biomolecular network with additional details. Incorporation of biomolecule age and the demand for biomolecule activities into a study of metabolism of multiple biomolecules will assist in investigations of the mechanism of dynamic changes at the biomolecule level. This subject requires further study, especially because it involves a huge amount of computational work.

**Reviewer #2**

**1) In this study, the authors proposed a model to explain the mechanism underlying intracellular RNA level fluctuations. The overall framework of the model seems to be logical and up to the task. I would like to raise some general points regarding this manuscript:**

Our response: Thank you for the comments.

**2) 1. Are there any references to support the definition of the age of a biomolecule used in this study?**

Our response: References cited in the present manuscript described the definitions of RNA age and half-life for RNA and protein (McManus et al., 2015; Xu and Asakawa, 2019; Rodriques et al. 2020). Numerous studies in molecular biology investigated the degradation and turnover of biomolecules, which are closely related to the age of these biomolecules. Thus, we defined the age of a biomolecule based on existing studies to better describe possible degradation and biological activity of a biomolecule at various time points after production.

[1] McManus J, Cheng Z, Vogel C. 2015. Next-generation analysis of gene expression regulation ‒ comparing the roles of synthesis and degradation. Mol Biosyst 11: 2680-2689.

[2] Xu Z, Asakawa S. 2019a. Modelling gene expression dynamics depending on activity demands and RNA age. In The Japanese Society of Fisheries Science spring meeting 2019-program and abstracts (ed. E Okazaki), pp. 69. The Japanese Society of Fisheries Science, Tokyo.

[3] Rodriques SG, Chen LM, Liu S, Zhong ED, Scherrer JR, Boyden ES, Chen F. 2020. Recording the age of RNA with deamination. bioRxiv doi: 10.1101/2020.02.08.939983.

**3) Page 18, the formula of degradation coefficient s should be provided.**

Our response: Thank you for the comment. The degradation coefficient, a parameter in the model, was used in the following equation:

$$B_{x,t}=B_{x-1, t-1}\times s_{x-1}$$

The first letter of the word “degradation” is “d”; hence, we used “d” instead of “s” to represent the degradation coefficient in the revised version.

**4) 3. R scripts for plotting should also provide**

Our response: Thank you for the comment. R scripts for plotting were added as supplemental materials in the revised version.

**5) 4. The Font in Figure 7 is too small to read. The authors are suggested to update this Figure**

Our response: Thank you for the comment. We have updated the figure to enlarge the font.

**6) 5. A brief description of the relationship between cellular protein levels and mRNA abundance is necessary, although it is summarised in the reported data (Liu et al., 2016)**

Our response: Thank you for the comment. A brief description of the relationships between cellular protein levels and mRNA abundance was added to the revised version as follows. The relationships between mRNA and protein abundance under various conditions is complex, and mRNA abundance is not sufficient to predict protein abundance in many scenarios. After excluding the influence of protein translation delay, three categories of representative relationships between RNA and protein levels were considered: cycling RNA levels and cycling protein levels, cycling RNA levels and stable protein levels, and stable RNA levels and stable protein levels.

**Reviewer #3**

**1)** **In this study, Xu et al. proposed a computational model with pre-defined parameters to explore the mechanisms of RNA fluctuation in living cells. The authors showed that the model fits well to the real experimental data and can explain RNA level fluctuations in response to different stimuli. Generally, the study provides some novel insights and looks interesting to me. However, I have the following concerns need to be addressed:**

Our response: Thank you for the comments.

**2)** **As my understanding, the authors fitted the parameters of their model using single-cell experimental data and showed that the parameters can predict values of the real experimental values well: “R^2^ of each gene was over 0.9, indicating that the model predicted a good fit with these experimental values (line103-104)”. First, what’s the R^2^ here mean? The authors should make it clear to the reader how they evaluate the consistency. Second, it is not clear how they fit the parameters; usually, one should fit the parameters in some data and see how it predict previous unseen data to show the performance. Here, I guess the parameters for different genes are different, so it is practical to leave some data points of certain gene out for cross-validation? If they just fit the parameters using all the data points and then predict the values, it’s not enough to make the statement “the model predicted a good fit with these experimental values”.**

Our response: Thank you for the comments. R^2^ is the coefficient of determination, which shows how well the model predicted the results, i.e., fit the real data. The equation for R^2^ is:

$$R^{2}=1-\frac{Unexplained variation}{Total variation}$$

To better illustrate whether the results of the model fit the experimental data, we added another coefficient, median absolute percentage error (MdAPE), to the revised manuscript to illustrate the quality of fit of the values predicted by the model and experimental values. MdAPE is the median of absolute percentage error, and the equation for absolute percentage error (APE) is:

$$\mathrm{APE}=\left| \frac{Actual value of experiment data-Prediction value of the model}{Actual value of experiment data} \right|$$

Our model may be applicable for prediction of unseen data, especially the data during the experiment period; however, the main goal of our model was to explain the mechanism of changes in RNA levels during the experiment. Thus, we used all data points for calculations. The parameters of the model are computational components that are also biologically significant. In the revised version, the sentence “R^2^ of each gene was over 0.9, indicating that the model predicted a good fit with these experimental values” was changed to “The calculated values of R^2^ and median absolute percentage error (MdAPE) of the model were a good fit for the experimental values**”.**

**3)** **The authors show three examples representing regular, partially regular and irregular fluctuations, which is good. However, to show the robustness of a computational model, it needs to be tested on data set at large scale. Considering that it may be difficult to perform a systematic evaluation in the current study, the author can at least provide one more example for each category in addition to the current one example. I found the data used in the study are from very old publications, is there any newer single-cell data available in larger scale?**

Our response: Thank you for the comments. We have added three examples as supplemental figure (Figure S1) in the revised version. The data used in the present study were obtained from previous publications. These data met the requirements of the study: 1) the experimental data described time series gene expression in single cells; and 2) according to Figure 2f, the sampling interval was shorter than the lifespans of RNAs. The RNA half-life in fungi can be less than 20 minutes, and the RNA half-life in mammalian cells can be only a few hours (McManus et al, 2015). In a study on fungi (Hao and O'Shea 2011), the interval of sampling of fungal gene expression in single cells was 10 minutes; in a study on mammalian cells (Nuñez et al. 1998), the interval of sampling of mouse gene expression in single cells was 30 minutes. At present, we have found suitable data only in a few references.

[1] Hao N, O'Shea EK. 2011. Signal-dependent dynamics of transcription factor translocation controls gene expression. Nat Struct Mol Biol 19: 31-39.

[2] McManus J, Cheng Z, Vogel C. 2015. Next-generation analysis of gene expression regulation ‒ comparing the roles of synthesis and degradation. Mol Biosyst 11: 2680-2689.

[3] Nuñez L, Faught WJ, Frawley LS. 1998. Episodic gonadotropin-releasing hormone gene expression revealed by dynamic monitoring of luciferase reporter activity in single, living neurons. Proc Natl Acad Sci USA 95: 9648-9653.

**4)** **In line 490-493, the authors mentioned that “We used the data provided by these studies to verify the outputs of model simulations”. Yet, no details are provided about how they verify the outputs using the experimental data.**

Our response: Thank you for the comment. In the text and figure legends in the revised version, we have indicated which experimental data can verify the corresponding simulation results. Figure 1b (Hao and O'Shea 2011) verified the output of “Cycles in fluctuations in RNA levels” (Figure 2); Figure 1c (Nuñez et al. 1998) and Figure S1c (Hao and O'Shea 2011) verified the output of “Environmental variations influencing fluctuations in RNA levels” (Figure 3), and Figure S1b (Hao and O'Shea 2011) verified the output of “Stable RNA levels” (Figure 4).

**5)** **Some figures, for examples, figure 2a, 3a, 4a, are hard to understand according to the figure legends, is it an example or just a schematic plot? The meaning of x-axis and y-axis is unclear. Further explanations about these figures in the legend are required. Fonts in figure 7a are too small.**

Our response: Thank you for the comments. Figures 2a, 3a, and 4a are schematic plots in Figures 2, 3, and 4, respectively; however, these panels also include the corresponding examples. Figure 2a corresponds to Figure 1b (Hao and O'Shea 2011); Figure 3a corresponds to Figure 1c (Nuñez et al. 1998) and Figure S1c (Hao and O'Shea 2011); Figure 4a corresponds to Figure S1b (Hao and O'Shea 2011). We have added a description of Figures 2a, 3a, and 4a in the revised manuscript. The descriptions of x-axis and y-axis have been added to the figure legends. A partially enlarged view has been added to Figure 7a to increase font size.

**6)** **The writings of the manuscript seem unbalanced, I found the “Discussion” part interesting to read while the “Results” part very descriptive and boring. I would suggest that the authors put some of their own interpretations after describing the data in the “Results” instead of putting them in the “Discussion”.**

Our response: Thank you for the comments. The revised version includes some interpretations after the description of the data in the “Results” section.

Sincerely,

Zhongneng Xu
